# The Role of Notch Signaling and Leptin-Notch Crosstalk in Pancreatic Cancer

**DOI:** 10.3390/medicines5030068

**Published:** 2018-07-02

**Authors:** Adriana Harbuzariu, Gabriela M. Oprea-Ilies, Ruben R. Gonzalez-Perez

**Affiliations:** 1 Department of Microbiology, Biochemistry and Immunology, Morehouse School of Medicine, Atlanta, GA 30310, USA; aharbuzariu@msm.edu; 2 Department of Pathology, Emory University, Atlanta, GA 30322, USA; goprea@emory.edu

**Keywords:** pancreatic cancer, Notch, RBP-Jk, leptin, chemoresistance

## Abstract

There is accumulating evidence that deregulated Notch signaling affects cancer development, and specifically pancreatic cancer (PC) progression. Notch canonical and non-canonical signaling has diverse impact on PC. Moreover, the actions of RBP-Jk (nuclear partner of activated Notch) independent of Notch signaling pathway seem to affect differently cancer progression. Recent data show that in PC and other cancer types the adipokine leptin can modulate Notch/RBP-Jk signaling, thereby, linking the pandemic obesity with cancer and chemoresistance. The potential pivotal role of leptin on PC, and its connection with Notch signaling and chemoresistance are still not completely understood. In this review, we will describe the most important aspects of Notch-RBP-Jk signaling in PC. Further, we will discuss on studies related to RBP-Jk-independent Notch and Notch-independent RPB-Jk signaling. We will also discuss on the novel crosstalk between leptin and Notch in PC and its implications in chemoresistance. The effects of leptin-Notch/RBP-Jk signaling on cancer cell proliferation, apoptosis, and drug resistance require more investigation. Data from these investigations could help to open unexplored ways to improve PC treatment success that has shown little progress for many years.

## 1. Introduction

PC is one of the most malignant and chemotherapy-resistant tumors. That is mainly due to the lack of effective diagnosis at an early stage of tumor development and ineffective therapy. Resectable tumors at detection time comprise a low percentage of PC. Majority of PC are treated with non-specific target therapies, i.e., chemotherapeutic drugs, but its effectiveness is limited due to the dense tumor stroma (desmoplasia) and the acquisition of drug resistance. Multiple mechanisms are involved in PC chemoresistance, including aberrant gene expression, deregulation of key signaling pathways, development of the epithelial-mesenchymal transition (EMT), cancer stem cells and desmoplasia [1].

A factor involved in cancer chemoresistance is the activation and deregulation of the Notch signaling pathway. Notch signaling has been recently linked to 5-FU resistance in PC [2]. This embryonic signaling pathway controls the maintenance and proliferation of the stem cell niche, cell fate decisions and can drive carcinogenesis [3]. The family of Notch receptors includes four transmembrane proteins (Notch1–4), which are activated upon interaction with different types of ligands [Serrate-like Jagged1 and 2 (JAG1–2) and Delta-like (Delta1–4)] expressed by adjacent cells. Notch signaling involves two sequential cleavage steps. Notch receptor binding to ligands induces the S2 site for cleavage by tumor necrosis factor-α-converting enzyme [4]. Next, γ-secretase catalyzes the truncation of Notch producing NICD (Notch Intracellular Domain). NICD binds to the nuclear recombination signal binding protein for immunoglobulin kappa J region (RBP-Jk) or CBF1/CSL (a key regulatory factor of Notch canonical signaling) that initiates the expression of several genes [5].

Notch target genes include Hes (hairy-enhancer of split), Hey (Hes related to YRPW), cell cycle and survival proteins (cyclin D1, p21, NF-κB, Myc), survivin and snail homolog 2 (SLUG) [4]. Notch signaling is required for pancreatic tissue formation and maintenance of undifferentiated epithelial cells. Activating germline mutations in Notch1, DLL1, Hes1 or RBP-Jk abrogate growth and branching of pancreas, deplete pancreatic epithelial precursor cells, and eliminate or delay the formation of islets and acini [6]. Comparable defects can be induced by the premature overexpression of the pro-endocrine *Ngn3* gene, which antagonizes Hes1 and suspends Notch control [7]. In contrast, low expression of activated Notch induces undifferentiation of progenitor cells of the pancreatic epithelium [8].

**Figure 1:**
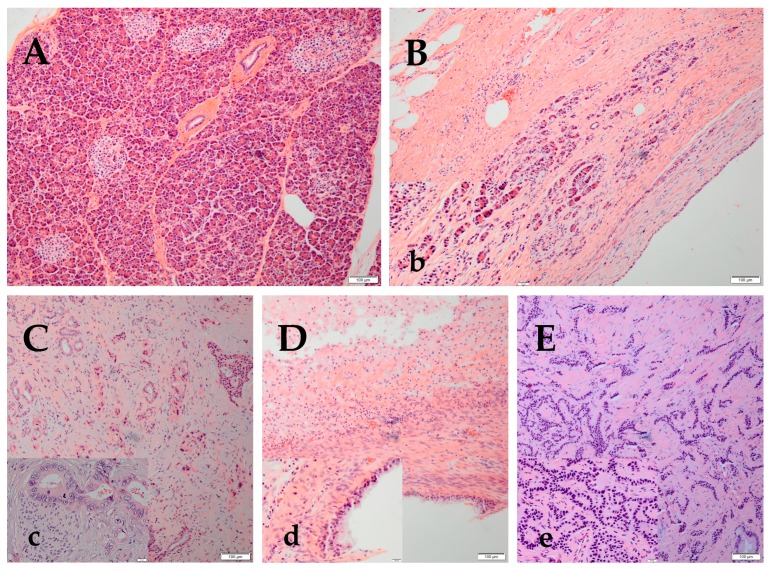
Representative pictures from hematoxylin and eosin staining of normal pancreatic parenchyma, chronic pancreatitis (CP), pancreatic adenocarcinoma (PA), mucinous pancreatic cyst and pancreatic neuroendocrine tumors (NET). (**A**) Normal pancreas shows acini and interlobular ducts (exocrine) and islets of Langerhans (endocrine) (10×) (**B**) CP shows loss of acini and ductal tissue, as well as periductal fibrosis (10×). (**b**) The thumb image is a lower magnification of CP, depicting residual islets and interlobular ducts with flattened epithelium (40×). (**C**) PA is composed of small glands and malignant cell clusters with hyperchromatic nuclei invading in a desmoplastic stroma (10×). (**c**) The high magnification (40×) of a moderately differentiated PA shows glands composed of tall columnar cells with abundant cytoplasm. Perineural invasion, one of the characteristics of PA, is also seen here. The tumoral nuclei are large, with irregular nuclear membrane, frequently vesiculated chromatin, with numerous chromocenters and occasional proeminent, cherry red nucleoli. (**D**) The pancreatic mucinous cyst is composed of cells which contain intracytoplasmic mucin and fibrosed stroma (10×). (**d**) The high magnification (40×) shows mucin secreting glandular cells lining a benign mucinous cyst of pancreas (40×). (**E**) NET is composed of cells forming trabeculae, cords and ribbons of neoplastic cells (10×). (**e**) The high magnification (40×) photo shows a well differentiated NET of the pancreas; the cells are small to medium in size, with eosinophilic to amphiphilic and finely granular cytoplasm. The nuclei are monotonous, uniform, eccentrically located, round-to-oval, with “salt and pepper” (finely stippled) chromatin and no conspicuous nucleoli.

The pancreas parenchyma has exocrine acini that secretes digestive enzymes, which flow through a ductal system, and an endocrine component (islets of Langerhans), which secretes insulin, glucagon and somatostatin (Figure 1A). Notch signaling is almost undetectable in the adult pancreas. However, in chronic pancreatitis (CP), the exocrine acinar cells lose their normal structure (Figure 1B) and show acino-ductal metaplasia, which significantly upregulates Notch1 and Notch2 expression and the target genes Hes1, and Hey1, and Hey2 [9]. Pancreatic adenocarcinoma (PA) (Figure 1C) is the most frequent of pancreatic malignant tumors (90% of PC cases). Notch signaling plays a major role in PA. Studies have shown that Notch1 and Notch3 were increased in PA tissues and in cell lines (HPAC and Panc-1) [10]. Furthermore, JAG2 induced PA metastasis both in vitro and in vivo [11]. Among pancreatic tumors, 1% are cystic. Mucinous or high risk pancreatic cysts require follow-up or surgery (Figure 1D). Pancreatic neuroendocrine tumors (NET) (Figure 1E) represent 3–5% of PCs with a 5-years survival rate of 42% [12].

Next-generation sequencing (NSG) allowed the detection of KRAS/GNAS variants, that characterize pancreatic mucinous neoplasms. Variants in TP53, SMAD4, CDKN2A, and Notch1 can identify a high-risk cyst [13]. Studies suggest a tumor suppressive function for Notch1 in neuroendocrine lineage cells [14]. Malignant insulinomas had no signs of Notch expression, in contrast to benign insulinomas. The missing Notch expression in the malignant pancreatic NET might be used as a potential predictive marker [15]. Furthermore, Notch1 activators could be novel pharmacological agents in some pancreatic NET [16].

In sharp contrast to normal pancreatic tissue, the activation of Notch signaling is linked to the development of PC. Notch signaling is important for initiation of precancerous pancreatic intraepithelial neoplasia or PanIN [17]. Furthermore, coactivation of KRAS and Notch resulted in PanIN, contributing to the development [18] and invasion of PC [19]. Additionally, Notch1 showed oncogenic actions in PC only when KRAS was coactivated [20]. It was early demonstrated that Notch inhibited differentiation in the embryogenic pancreas and in tumor-initiating cells. Overall, Notch and KRAS exhibited a strong synergy in inducing PanIN formation [21]. Furthermore, PanIN shows high expression of Hes1 [17] and PC overexpress Notch pathway components [22].

Notch’s effects as tumor suppressor or oncogene vary depending on the tissue and cell type, and the degree of activation [23]. High expression of Notch1 and JAG1 correlate with poor prognosis in lung and breast cancer [24,25]. Notch1 is a suppressor of skin cancer. Moreover, Notch2 may have similar role in breast cancer [26,27]. The loss of Notch1 alleles leads to the development of spontaneous skin basal carcinoma in mice [27]. Notch1 is a very large gene (34 exons) that shows several inactivating mutations, suggesting it may function as a tumor suppressor gene in squamous cell carcinomas of the head and neck (HNSCCs) [28]. Notch also acts as a tumor suppressor in prostate, liver, and small cell lung cancer [23]. However, Notch oncogenic signaling have been reported in leukemia, breast, colorectal, cervical, lung and PC [29].

## 2. Notch Canonical and Non-Canonical Signaling

**Figure 2:**
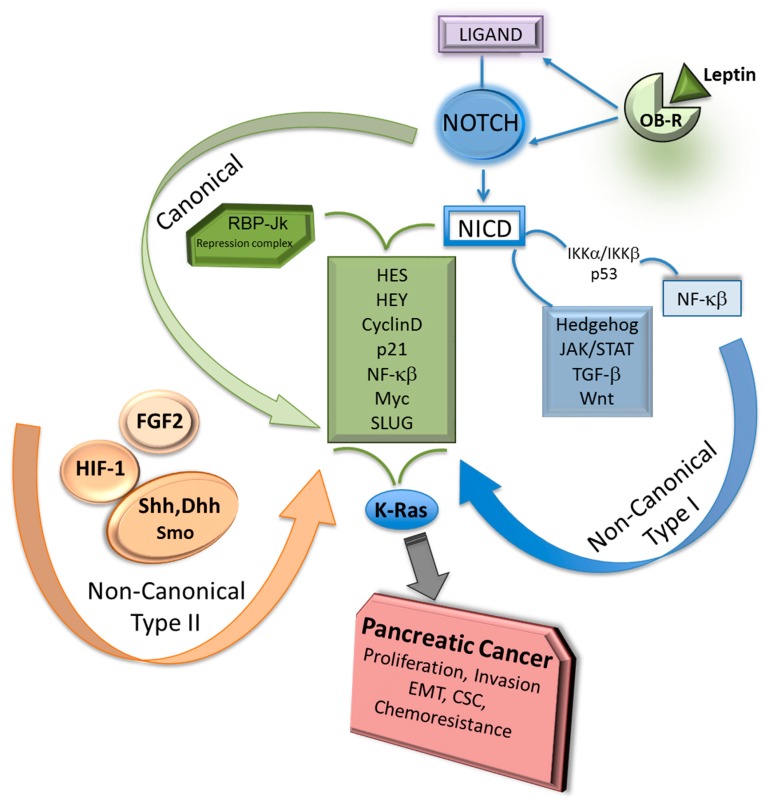
Notch canonical and non-canonical signaling pathways and PC. Notch canonical signals require the activation of membrane-bound Notch receptors via ligand binding and production of Notch intracellular domain (NICD), which binds to RBP-Jk repressor complex to induce transcription of Notch targets (Hes, Hey, Cyclin D, etc.) [4,5,18,20,22]. Notch non-canonical Type I signals require NICD involvement to activate NF-κB, Hedgehog, JAK/STAT. TGF-β or Wnt. In contrast, Notch non-canonical Type II signals triggered by diverse factors (i.e., FGF2, HIF-1α, Hedgehog) do not require NICD or RBP-Jk involvement [30,31,32,33,34,35,36]. Notch and KRAS activation show synergic effects to induce PC development and chemoresistance [21]. Leptin binding to its receptor induces the expression of Notch in PC [37] Shh: Sonic Hedgehog; Dhh: Desert Hedgehog; Smo: Smoothened protein; OB-R: leptin receptor.

Non-covalent binding between Notch and ligands triggers the canonical signaling upon the production of NICD that binds RBP-Jk in the nucleus (Figure 2). 

Notch canonical signaling is required by many normal cellular processes. Moreover, Notch-RBP-Jk canonical signaling is involved in pancreatic development, but also plays a role in PC progression. In contrast, cancer and activation of the immune system are linked to Notch non-canonical signaling [38].

Notch non-canonical signaling proceeds without RBP-Jk participation. There are two types of Notch non-canonical signaling (targeting gene activation via RBP-Jk independent Notch signals): Type I involves ligand-mediated activation of Notch receptors that transduces the pathway independent of RBP-Jk, and Type II, which involves the activation of Notch target genes completely unrelated to either Notch receptors cleavage or RBP-Jk mediated-signal transduction [39]. For example, Notch4 canonical signaling is required for the development of mammary glands, but Notch4-independent RBP-Jk signaling is related to mammary tumorigenesis. It is known that γ-secretase inhibitors (GSI) are not completely effective in blocking tumor-related Notch functions. This could be due to the effects of Notch non-canonical signaling [40]. Additionally, cytoplasmic and nuclear NICD signaling independent of RBP-Jk could influence malignant progression.

Notch non-canonical signaling pathways have been related to cell proliferation, neoplastic transformation, tumor progression and apoptosis [41,42]. Moreover, early neuron differentiation and B-lymphocyte lineage commitment are affected by RBP-Jk independent Notch signaling [43]. Notch1 and Notch2 share the highest structural homology in PC. Data from a PC cell line (BxPC-3) show that Notch1, Notch2 and RBP-Jk bind to different target genes. Few Notch1, Notch2 and RBP-Jk binding sites overlap, but approximately 50% of RBP-Jk binding overlap with Notch1 and Notch2, indicating that most of Notch signaling activities are RBP-Jk independent [30].

Active Notch can be also found in the endosomal compartment, where it may stimulate downstream targets. Activated Notch is directed to endosome by RING-finger protein Deltex, where it could induce RBP-Jk independent signals. Thus, subcellular compartmentalization of Notch may affect its access to specific targets [31].

Transcription factors and other embryonic signaling pathways could activate Notch non-canonical signaling Type II (Figure 2). Hes1 is the principal Notch target that can be activated via Type II signaling independent of Notch. Although there are scarce data on the role of Notch non-canonical signaling Type II in PC, accumulating evidence indicates that this specific signaling impacts several cell types including stem cells. It was earlier reported that BIRC3 mRNA expression correlated to Shh/Gli1 activation in PC cell lines. Shh induces survival and Gli1 binds to enhancer elements of BIRC3 promoter inducing gene transcription and PC cell proliferation [44]. Moreover, inhibition of Gli signaling sensitized PC cells to gemcitabine [45]. Conversely, Notch target Hes1 was described as a modulator of Hedgehog signaling and inducer of chemoresistance in medulloblastoma cells [46]. However, it is unknown whether Hedgehog-inducing effects could modify Notch signaling in PC.

FGF2 transactivates Hes1 expression independent of RBP-Jk and Notch in neural progenitors [43]. Additionally, Sonic Hedgehog induces Notch-independent Type II signaling, by regulating Hes1 expression in C3H/10T1/2 embryonic mesodermal and MNS70 in neural stem cells. Particularly, Smoothened function upregulates Hes1 in response to Sonic Hedgehog and Desert Hedgehog that can elicit a strong response in these cell lines. These Hedgehog’s mechanisms do not require γ-secretase mediated-cleavage of Notch receptors and involve transcription factors other than RBP-Jk. These factors include modulators of Wnt, IGF and TGF-β [47]. Moreover, Notch non-canonical signaling crosstalk with Hedgehog, JAK/STAT, TGF-β and Wnt pathways to control tissue organogenesis and homeostasis [35] and has important roles as cellular and developmental regulator through Wnt/β-catenin signaling [48]. Furthermore, ligand-independent Notch activation can occur through HIF-1α in *Drosophila* immune cells. Moreover, activation of the T-cell receptor leads to NICD production that may occur in the absence of ligand [38]. Similarly, Notch3 non-canonical signaling regulates T-cell development and leukemia through direct interaction with IKKα [32], suggesting that NF-κB pathway plays an important role in Notch non-canonical signaling related to oncogenesis.

Nuclear localization of NICD independent of RBP-Jk was linked to neoplastic transformation of baby rat kidney cells [49]. Additionally, NICD interaction with the NF-κB pathway in the cytoplasm induced IL-6 expression via p53 and IKKα/IKKβ axis in mammary cancer cells [50]. Whether Notch non-canonical signaling might exert comparable effects in PC needs to be investigated.

## 3. Notch-Dependent and Notch-Independent RBP-Jk Signaling

RBP-Jk or CSL/CBF1 in mammals and its orthologous proteins Su (H) in *Drosophila* and Lag-1 in *C. Elegans* have been identified as the main nuclear signaling partners for NICD [35]. RBP-Jk is a tumor suppressor and Notch signaling gate-keeper. When NICD is not present, RBP-Jk forms a complex composed of several transcriptional corepressors, thereby, it acts like a transcriptional switch [51]. The interaction of NICD with RBP-Jk induces the replacement of corepressors (Silencing mediator of retinoid and thyroid hormone receptors, SMRT; Ski-interacting protein; histone deacetylases, HDACs; CBF1 interacting corepressors, CIR; SAP30) [52] by the recruitment of activators such as the Mastermind-like family of proteins (MAML1–3) and histone acetyltransferase p300.

Notch-interacting protein, NACK, is a Notch transcriptional co-activator that associates with the NICD-MAML1-RBP-Jk transcriptional activation complex on DNA and mediates Notch1 transcriptional activity. NACK kinase activity has not been demonstrated yet. However, NACK has been suggested to be required for Notch1-mediated tumorigenesis and development, and its expression is Notch-dependent that establishes a feed forward loop in normal pancreas, PA and several cancer cell lines (i.e., esophageal, breast, T-cell acute lymphoblastic leukemia or ALL) [53]. NICD-RBP-Jk interaction promotes the expression of Notch target genes that are involved in proliferation, differentiation, survival and cell motility [54].

RBP-Jk canonical pathway signals in cancer are dependent of Notch. However, in the absence of NICD, RBP-Jk can recruit transcriptional co-repressors inhibiting the transcription of specific target genes [55]. Knockdown of RBP-Jk expression decreased proliferation of breast and prostate cancer cells, but GSI treatment induced minimal growth effects, further suggesting that RBP-Jk has Notch-independent roles [56]. In contrast to Notch1, RBP-Jk expression is frequently reduced in lymphoma, breast, brain, cervix, kidney, oral, lung, prostate and skin cancers. Also, RBP-Jk is lost in approximately 25% of PC [57]. That suggests RBP-Jk is an important tumor suppressor in some PC types.

RBP-Jk knockout accelerated breast cancer growth, indicating that it acts as a tumor suppressor independent of Notch. Moreover, loss of RBP-Jk induced the acquisition of stem cell-like phenotype, upregulation of Notch target genes [58] and enhanced tumor cell survival that was associated with Myc and NF-κB [56]. Reports from investigations in cardiomyocytes suggest that Notch-dependent RBP-Jk signaling repress the production of pro-angiogenic and angiostatic factors. In fact, RBP-Jk binds to HIF-1α and HIF-2α to control gene expression of angiogenic factors. Moreover, RBP-Jk deletion in adult cardiomyocytes increased micro vessel density, conferred tolerance to hypoxia and protected the heart from ischemic injury. Surprisingly, contrary to expectations, with RBP-Jk present, the activation of Notch did not induce angiogenic changes [59]. RBP-Jk can statically bind DNA without the recruitment of NICD or p300 in mouse myogenic cells, suggesting additional roles of RBP-Jk in controlling gene expression irrespective of Notch activity [52]. However, the role of Notch independent RBP-Jk signaling in PC is largely unknown.

## 4. Notch Pathway Mutations

Activating Notch mutations were early identified and implicated in T-cell ALL. This disease is characterized by NICD formation and increased stability by ligand-independent proteolytic cleavage of Notch1 [60].

Genetic alterations of Notch pathway components have also been observed in human solid malignancies. Most genetic alterations of Notch receptors have been observed in the Notch1 gene. Activating Notch1 mutations have been found in lung cancer. Several cancers (i.e., melanoma, colorectal carcinoma, cholangiocarcinoma) show Notch activation [61]. Epigenetic context could influence Notch mutations. A few independent research groups reported that up to 15% of HNSCC carry Notch1 mutations. Notch1 is not a therapeutic target because restoring action of loss-of-function genes is difficult, therefore, for these patients, these findings are clinically relevant. Another study found mutations and amplifications that activated Notch pathway in triple negative breast cancer and induced the expression of canonical Notch target genes [62]. Moreover, in mice, basal cell carcinoma-like and squamous cancers can be produced by loss of Notch activity [63].

In contrast to findings reported in other cancer types, genetic analysis of PC showed that Notch mutations are relatively rare, but several components of this pathway appear to be amplified, which was consistent with overexpression and deregulation of Notch pathway in PC [64].

## 5. Notch Pathway and PC Chemoresistance

Several studies show that various mechanisms can contribute to intrinsic and acquired resistance in PC. An important factor for the development of acquired drug resistance is a refractory tumor environment. Indeed, PC tissue is characterized by the development of desmoplasia that is linked to the proliferation of cancer associated fibroblasts and deposition of extracellular matrix. Desmoplasia reduces tissue elasticity, increases interstitial fluid pressure, and decreases therapeutic agent rate of perfusion and efficacy [65].

PC chemoresistance can also involve changes in the expression levels of ATP-cassette of efflux proteins (ABC family), and activation of oncogenic pathways and processes: HIF, survival factors (NF-ĸB, PI-3K/Akt)], EMT (epithelial-mesenchymal-transformation) and PC stem cells (PCSC) [66].

The expression of ABC family of proteins impairs the effects of chemotherapeutics by increasing the efflux of drugs from the cells. Several ABC transporters were upregulated in PC compared to non-neoplastic tissue [67]. Additionally, ABC proteins were upregulated by Notch pathway in medulloblastomas [68]. Similarly, in MCF-7 breast cancer cells, Notch1 canonical signaling upregulated the expression of ABCC1 protein [69].

EMT induces higher migratory, invasive, anti-apoptotic and extracellular matrix degradation capacities that characterized the mesenchymal phenotype. EMT was associated with overexpression of Notch signaling components that correlated to higher migration and invasion of PC cells. Notably, EMT phenotype was reverted via Notch inhibition, which reduced the expression of ZEB1, Snail, Slug and Vimentin in PC cell lines [70,71]. Moreover, Notch signaling induced EMT, increased CD44+ PCSC, aggressive tumor behavior and resistance to conventional chemotherapy [72]. Additionally, the Notch pathway was involved in the acquisition of the mesenchymal phenotype of PC gemcitabine-resistant cells [70]. EMT phenotype in gemcitabine-resistant PC cells correlated to Notch2, Notch4, and JAG1 overexpression [73]. Suppression of Notch activity and impairment of NF-κB signaling reduced PC cells invasiveness [74].

DCAMKL-1 is a microtubule-associated kinase that downregulates pluripotent cancer stem cell markers (Snail, Slug and Twist), but induces microRNA200 (miRNA200a). DCAMKL-1 was overexpressed in a subset of PanIN and PC cells. EMT was inhibited via knockdown of DCAMKL-1 that also inhibited Notch1 via miR-144 [75].

The Notch signaling pathway is a component of the stem cell signaling network (SCSN) together with Wnt, FGF, BMP and Hedgehog signaling pathways [76]. SCSN is implicated in embryogenesis and maintenance of adult tissue homeostasis. Deregulation of SCSN leads to pathological conditions, such as congenital disorders, metabolic syndrome, and cancer. The activation of Notch canonical signaling results in the maintenance of stem or progenitor cells through the inhibition of differentiation [77].

Cancer stem cells are commonly more resistant to chemotherapeutics, which preferably target the bulk of the tumor. Therefore, chemotherapeutic actions spare stem cells, which show higher expression of ABC proteins, detoxification proteins and survival pathways (NF-κB and PI-3K) and diminished apoptosis rate [78]. Earlier reports showed that Notch signaling pathway is essential for self-renewal of stem cells and cell-fate determination of progenitor cells [79,80,81,82]. PC cells expressing stem cell markers CD44+CD133+ showed higher expression levels of Notch [83]. PCSC overexpress Notch1 and Notch2 compared to normal pancreatic cells [84]. It was reported that PCSC overexpressing pluripotent factors (Oct-4, Sox2, NANOG) have enhanced aggressiveness and marked chemoresistance [85]. Moreover, DLL4 expression in PC cells increased Oct-4 and Nanog that expanded PCSC populations [86].

A wide-used PC drug, gemcitabine, increased PCSC expressing CD24+ and CD133+, stemness-associated genes (Bmi1, Nanog, and Sox2), cell migration, chemoresistance, and tumorigenesis [51]. Gemcitabine induced PCSC overexpressing ABCB1 and CD44+ that correlated with higher tumor histological grade and worse prognosis [3]. Moreover, Notch2, Notch4 and JAG1 overexpression correlated with PC-Gemcitabine chemoresistance [70]. Additionally, Notch4 overexpression was linked to PC-Docetaxel chemoresistance [87].

MicroRNAs (miRNA or miR) are noncoding endogenous RNA of 14-24 nucleotides that can regulate protein expression at the post-transcriptional level. Many studies have found strong correlations between deregulated miRNA and cancer. Decreased expression of microRNA (miRNA34 and miR200) families was early associated with PC progression and PCSC [88]. It has been demonstrated that p53 regulates miR-34, which acts as a down regulator of the Notch and Bcl-2 pathways. Indeed, miR-34 levels were reduced in MiaPaCa-2 PC cells that expressed CD44+CD133+ stem cell markers. MiR-34 restoration reduced stem cells, in vitro tumorspheres growth and PC formation [84]. Notch inhibition reduced stem cell markers CD44 and CD24 in gemcitabine-treated PC cells [89]. In line with these results, the inhibition of Notch signaling depleted ALDH+ PCSC [90].

MiRNA200 family (miR200a,b,c), miR429, and miR141 have been implicated in PC chemoresistance. miR200c expression correlated with chemoresistance and less cancer invasion via repression of cancer stem cell self-renewal and differentiation, inhibition of EMT and attenuation of apoptosis. In PC, miR200 repressed self-renewal and differentiation of stem cells, and inhibited EMT by interacting with ZEB1/2 and the Notch pathway [91]. Moreover, knockdown of miR21 inhibited PC cell proliferation and tumor growth. We recently reported a preliminarily analysis of miR21 and miR200 expression in PC biopsies using TCGA databank, which showed PC progression correlates to higher expression of miR21 when compared with miR200 [88].

## 6. Notch Pathway and Adipokines in PC

Adipokines (leptin and adiponectin) are adipose tissue-derived cytokines that can act in opposing manner: leptin contributes to a pro-tumorigenic phenotype, while adiponectin acts as anti-tumorigenic factor. The pluripotential actions of leptin as an inflammatory, mitogenic and proangiogenic factor, have been linked to cancer cell proliferation, recurrence, tumor angiogenesis and chemoresistance. We firstly reported that leptin induces the expression of Notch family components in breast cancer, which was linked to IL-1 signaling [72,92,93]. Leptin also induces the expression of Notch receptors and ligands in PC [37] (see Figure 2). A novel and complex signaling crosstalk between leptin, Notch and IL-1 (Notch, IL-1 and leptin crosstalk outcome, NILCO) seems to drive leptin-induced oncogenic actions in breast cancer. NILCO could represent the integration of developmental, pro-inflammatory, and pro-angiogenic events critical for leptin-induced cell proliferation/migration and tumor angiogenesis [91].

RNA knockdown and pharmacological inhibitors of leptin signaling significantly abrogated the activity of reporter gene-luciferase CSL (RBP-Jk) promoter, showing that its activity was linked to JAK2/STAT3, MAPK, PI-3K/mTOR, p38 and JNK signaling pathways. DAPT (a GSI) and siRNA RBP-Jk abrogated leptin’s effects on cell proliferation/migration, Notch, IL-1 and VEGF/VEGFR-2 [92]. Furthermore, Notch loss-of-function (via DAPT-induced Notch inhibition) and the expression of dominant negative [R218H] RBP-Jk [CSL/CBF1]) impaired leptin-induced cell proliferation and migration. Moreover, diet-induced obesity, which increased leptin levels and signaling in mice hosting mammary tumors, incremented the levels of Notch3, JAG1 and survivin [93]. In vivo leptin signaling inhibition reduced Notch and target expression (NICD1, NICD4, Notch3, JAG1 and survivin), suggesting that leptin-Notch crosstalk could be involved in the reported higher incidence, aggressiveness and poor prognosis of breast cancer in obese patients [72]. Additionally, leptin and Notch signaling seem to crosstalk in endometrial cancer. The analysis of endometrial cancer (EmCa) biopsies showed that NILCO (Notch1–4, ligands, IL-1/IL-1R, leptin and Ob-R) molecules were expressed higher in type II EmCa. In addition, leptin-induced cell invasion of EmCa cells was abrogated by the inhibition of NILCO [94]. Thus, NILCO could be a key link between obesity and cancer progression [95].

It is known that leptin and Notch signaling mediate the activation of cancer stem cells that can affect drug resistance [3,54,95]. We have further shown that leptin and its receptor OB-R are expressed on PC cells, establishing an autocrine/paracrine signaling loop [37]. Moreover, in PC cells, leptin induced the expression of Notch receptors, ligands and target molecules (Notch1–4, DLL4, JAG1, survivin and HEY2), PC stem cell markers (CD24/CD44/ESA, ALDH, CD133, Oct-4), ABCB1 (P-glycoprotein 1 or MDR1 or CD243). In addition, leptin increased PC tumorsphere formation, cell cycle progression, proliferation, and tumorigenesis. These effects were reduced by DAPT [37]. It was also reported that mouse and human pancreatic tumors and PC cell lines showed reduced levels of adiponectin receptors in comparison to normal pancreatic tissue. Additionally, treatment of PC with adiponectin or an adiponectin receptor agonist, AdipoRon, suppressed leptin-induced STAT3 signaling in vitro and reduced tumor growth [96].

Our published data also show that leptin is a survival factor for PC cells treated with chemotherapeutics. Interestingly, 5-FU’s toxic effects on PC cells were impaired by leptin. PC cells treated with 5-FU in presence of leptin showed significantly higher proliferation rate and colony forming ability. In addition, these cells expressed higher levels of EMT, pluripotency and PCSC markers. Notably, leptin increased the expression of ABC proteins (ABCC5 and ABCC11), and Notch in PC cells treated with 5-FU. Moreover, leptin impaired 5-FU-induced apoptotic effects by increasing RIP and Bcl-XL and reducing Caspase-3 activation, PARP degradation and Bax expression. Remarkably, these leptin effects on PC cells treated with 5-FU were dependent of Notch signaling [2]. In addition, the number and size of PC tumorspheres treated with 5-FU and leptin were reduced by iron oxide nanoparticle-bound LPrA2 (IONP-LPrA2), a leptin signaling inhibitor [2]. These results could be clinically relevant, suggesting that adipose tissue and tumor cells secrete leptin that can desensitize cancer cells to chemotherapeutics, which could further contribute to the dismal prognosis of PC patients.

## 7. Targeting Notch Signaling in PC

The Notch signaling pathway can be inhibited pharmacologically, via GSI, anti-Notch antibodies, etc. GSI have been tested in clinical trials for many cancers, including PC. The agent RO4929097 in combination with gemcitabine was safely tolerated, achieved clinical antitumor activity and more than four months stable disease in PC, tracheal, and breast cancers [97]. A selective GSI, PF-03084014, induced PC xenografts regression by targeting stem cells [98]. Dual treatment of GSI IX and JAK2 inhibitor (AG-490) dramatically impaired growth and invasion of human PC cell and attenuated tumor progression in vivo compared to monotherapy [99]. However, the use of GSI has several limitations, as these drugs could have unwanted cytotoxicity in the gastrointestinal tract and could impair the cleavage of γ-secretase substrates in addition to Notch [100]. MRK-003, a GSI, induces downregulation of nuclear Notch1 intracellular domain (N1ICD) and reduces PCSC. MRK-003 and gemcitabine combined treatment showed enhanced effects on PC compared to gemcitabine alone, reduced tumor cell proliferation and induced both apoptosis and intra-tumoral necrosis [101]. MK-0752, a potent oral GSI, administered in a Phase I clinical trial in combination with gemcitabine, induced PC stable disease in 13 of 19 patients and partial response in 1 patient [102].

PC has also been treated with monoclonal antibody-based therapies. A fully humanized anti-Notch2 and Notch3 antibody (Tarextumab or OMP-59R5) reduced the growth of PC xenografts in mice when combined with cytotoxic drugs. Tarextumab (Phase2 trial 9NCT01647828) in combination with gemcitabine and nab-paclitaxel (Abraxane) was applied to untreated metastatic PC patients. The monoclonal antibody-based therapy allowed 5.6 months of median time of progression-free survival and 11.6 months of overall survival. PC patients with high levels of Notch3 showed better response [103]. In another clinical study, PC patients with locally advanced or metastatic disease received Demcizumab (humanized IgG2 anti-DLL4 antibody) and gemcitabine/nab-Paclitaxel (Abraxane). However, the clinical trial did not meet the primary end point of progression-free survival [104]. To overcome GSI unwanted effects, Quinomycin (a quinoxaline antibiotic that was originally isolated from Streptomyces echinatus) has been used to inhibit Notch pathway in PC [105]. Additionally, Genistein (a natural isoflavone primarily found in soybeans and soybean-enriched products) has also been used to suppress Notch1 expression in PC cells. Genistein inhibited cell growth, migration, invasion, EMT phenotype, formation of tumorspheres and induced apoptosis in PC [106] (see Table 1).

Recent reports suggested that inhibitors of other components of Notch signaling (i.e., Notch-activation enzymatic cascade and Notch transcriptional activation complex), could open new targeted therapy opportunities. Indeed, the inhibition of ADAM10 (overexpressed in PC) via a calcium channel blocker (Fendiline) significantly reduced proliferation and tumorigenesis of PC cells [107]. Additionally, a small molecule IMR-1 (inhibitor of Mastermind Recruitment-1) that disrupts the formation of the NICD1-MAML1-RBP-Jk transcriptional activation complex, was used to inhibit Notch signaling in esophageal, lung, breast and human fibrosarcoma cancer cell lines. Results from these studies suggested that IMR-1 could be a new promising anti-Notch therapy for cancer [108].

Obesity, characterized by high levels of leptin, is a PC risk factor [109]. In view of the many effects shown by leptin on PC chemoresistance, cancer stem cells and Notch and RBP-Jk signaling, we speculate that the inhibition of leptin signaling might be a new strategy to sensitize PC to chemotherapeutics [54].

Pre-clinical data from our laboratory have shown a potential for leptin signaling inhibition (i.e., using IONP-LPrA2) as adjuvant for chemotherapeutic treatment of PC [3,37,52,96,97]. Other studies have shown that the theranostic IONP-Gemcitabine (IONP-Gem) nanoparticles have great potential for the development of targeted therapeutic and imaging approaches that can overcome the tumor stromal barrier, thus enhancing the therapeutic effect of nanoparticle drugs on PCs [110]. As a result, future investigations using theranostic IONP-Gemcitabine particles coupled with leptin antagonists could be a novel approach in PC treatment. These considerations could be paramount to establish new therapies for obese patients, which show higher levels of leptin and PC risk [54].

## 8. Conclusions

PC dismal survival and poor treatment outcomes have been steady over years. Reported data underline the role of Notch in PC and other cancers development. However, the activation and regulation of Notch signaling in PC are still not completely understood. The specific contribution and regulation of Notch canonical and non-canonical signaling as well as the less investigated RBP-Jk signaling independent of Notch to oncogenesis and progression of PC remain scientific challenges. Leptin is a factor that could affect Notch signaling. Leptin’s levels are increased in obesity that is pandemic and strongly linked to incidence of PC and other cancers. Analysis of recent data shows strong relationships between leptin and Notch/RBP-Jk signaling that could open new opportunities of research and potentially increase the success of PC treatment and decrease chemoresistance. The crosstalk between leptin, Notch and RBP-Jk signaling in PC warrants further investigations.

## Figures and Tables

**Table 1:** Notch targeted therapy in PC.

Class	Drug	Target	Study type	Results	Reference
GSI	R04929097	γ-secretase	Clinical trial	>4 months stable disease	Yuan X Cancer Letters, 2015 [97]
	PF-03084014		Pre-clinical	Reduces PCSC, xenograft growth	Yabuuchi S Cancer Letters, 2013 [98]
	MRK-003		Pre-clinical	Induces apoptosis and tumor necrosis	Mizuma M Carcinogenesis, 2013 [101]
	MK-0752		Clinical trial	68% of patients achieved stable disease	Cook N Br J Cancer, 2018 [102]
GSI+JAK2 inhibitor	GSI IX + AG-490	γ-secretase +JAK2	Pre-clinical	Suppresses the conversion of acinar-ductal metaplasia to PC	Palagani V Carcinogenesis, 2013 [99]
Monoclonal antibody	Tarexumab	Notch2, Notch3	Pre-clinical	Reduces tumor xenograft growth	Knudsen ES Gastroenterology, 2016 [103]
			Clinical trial (+Gemcitabine, nab-Paclitaxel)	5-6 months PFS11.6 months OS	
	Demcizumab	DLL4	Clinical trial (+Gemcitabine,Nab-Paclitaxel)	No difference to chemotherapy	Gracian AC Annals of Oncology, 2017 [104]
Other treatments	Quinomycin	Antibiotic	Pre-clinical	Reduces PCSC and tumor growth	Ponnurangam S Oncotarget, 2016 [105]
	Genistein	Isoflavone	Pre-clinical	Reduces apoptosis through upregulation of miR-34a	Xia J Curr Drug Targets, 2012 [106]
	Fendiline	ADAM10	Pre-clinical	Reduces cell proliferation, migration and PCSC	Woods N Oncotarget, 2015 [107]
	IMR-1	Mastermind Recruitment-1	Pre-clinical	Disrupts the formation of NICD1-MAML1-RBPJ activation complex	Astudillo L Cancer Research, 2016 [108]
	IONP-LPrA2	OB-R	Pre-clinical	Reduces PC xenograft growth and re-sensitizes PC cells to chemotherapy	Harbuzariu A Oncotarget, 2017 and 2018 [2,37]

PFS: progression free survival; OS: overall survival; OB-R: leptin receptor; GSI: γ-secretase inhibitor.
